# TaqMan probe assays on different biological samples for the identification of three ambrosia beetle species, *Xylosandrus compactus* (Eichoff)*, X. crassiusculus* (Motschulsky) and *X. germanus* (Blandford) (Coleoptera Curculionidae Scolytinae)

**DOI:** 10.1007/s13205-021-02786-9

**Published:** 2021-05-10

**Authors:** Domenico Rizzo, Daniele Da Lio, Linda Bartolini, Chiara Salemi, Dalia Del Nista, Antonio Aronadio, Fabrizio Pennacchio, Francesco Binazzi, Valeria Francardi, Antonio P. Garonna, Elisabetta Rossi

**Affiliations:** 1 Laboratory of Phytopathological Diagnostics and Molecular Biology, Plant Protection Service of Tuscany, Via Ciliegiole 99, 51100 Pistoia, Italy; 2 Department of Agricultural, Food and Agro-Environmental Sciences, University of Pisa, Via del Borghetto 80, 56124 Pisa, Italy; 3 Laboratory of Phytopathological Diagnostics, Plant Protection Service of Tuscany, via delle Colline, Collesalvetti, 57014 Livorno, Italy; 4 CREA—Research Centre for Plant Protection and Certification, via Lanciola 12/A, 50125 Florence, Italy; 5 Department of Agricultural Sciences, University of Naples Federico II, 80055 Portici, Italy

**Keywords:** Black twig-borer, Granulate ambrosia beetle, Black timber bark beetle, Molecular diagnostics

## Abstract

Molecular assays based on qPCR TaqMan Probes were developed to identify three species of the genus *Xylosandrus*, *X. compactus, X. crassiusculus* and *X. germanus* (Coleoptera Curculionidae Scolytinae). These ambrosia beetles are xylophagous species alien to Europe, causing damages to many ornamental and fruiting trees as well as shrubs. DNA extraction was carried out from adults, larvae and biological samples derived from insect damages on infested plants. For *X. compactus*, segments of galleries in thin infested twigs were cut and processed; in the case of *X. crassiusculus*, raw frass extruded from exit holes was used, while DNA of *X. germanus* was extracted from small wood chips removed around insect exit holes. The assays were inclusive for the target species and exclusive for all the non-target species tested. The LoD was 3.2 pg/µL for the frass of *X. crassiusculus* and 0.016 ng/µL for the woody matrices of the other two species. Both repeatability and reproducibility were estimated on adults and woody samples, showing very low values ranging between 0.00 and 4.11. Thus, the proposed diagnostic assays resulted to be very efficient also on the woody matrices used for DNA extraction, demonstrating the applicability of the protocol in the absence of dead specimens or living stages.

## Introduction

The subfamily Scolytinae (Coleoptera: Curculionidae) includes more than 6000 species worldwide divided into 26 tribes and 247 genera (Vega and Hofstetter 2015). Scolytinae are xylophagous beetles colonizing the wood at phloem (phleophagous species) or xylem level (xylomycetophagous species) of many ornamental and forest plants (Kirkendall and Biedermann 2015). In recent years, Europe and the Americas have been affected by the introduction and establishment of numerous exotic species of the Xyleborini tribe, which caused growing concerns for native biodiversity and forest resources (Kirkendall and Faccoli 2010; Grousset et al. 2020). Their ability to escape phytosanitary controls due to their cryptobiotic behaviour, their capacity to establish in the new environment due to their reproductive behavior, their large polyphagy and the increasing global trade of wood (Reed and Muzika 2010; Rassati et al. 2016a; Smith et al. 2020) make Xyleborini a potentially invasive taxon.

The genus *Xylosandrus* Reitter, 1913, is a large genus of the Xyleborini ambrosia beetles presently including 40 species widespread in tropical and temperate areas (Dole and Cognato 2010; Dole et al. 2010). In Europe and North America, *X. compactus*, *X. crassiusculus* and *X. germanus* have been listed among the species of major concern (Rassati et al. 2016b).

The first report for Europe of *X. compactus* (the shot-hole borer or black twig-borer) dates back to 2012, in urban parks of the Campania and Tuscany regions (Italy) (Garonna et al. 2012; Pennacchio et al. 2012a) but its current distribution in Italy has further expanded (Francardi et al. 2017). To date, the species is established in France, Greece as well as Monaco and it is currently under eradication in Spain (CABI 2020; EPPO 2020). *X. compactus* shows a highly invasive potential and represents a serious threat in the Mediterranean Basin due to its wide host range and multitrophic interactions (Vannini et al. 2017; Gugliuzzo et al. 2019; Contarini et al. 2020).

As ambrosia beetle, it is associated with the primary symbiont *Ambrosiella xylebori* (Bateman et al. 2016; Vannini et al. 2017; Gugliuzzo et al. 2020) and with non-mycangial fungi such as *Fusarium solani* (Bosso et al. 2012), canker and dieback pathogens of woody hosts (Gugliuzzo et al. 2020) and several other fungal species (Morales-Rodriguez et al. 2021).

*Xylosandrus crassiusculus* (the granulate ambrosia beetle) is a polyphagous species, native to tropical and subtropical regions of Asia, but now present in some European countries (limited areas of France, Italy, Slovenia, Spain), Africa, American continent, and Oceania (Pennacchio et al. 2003; CABI 2019a; EPPO 2020). The host plants include many forest and ornamental woody species as well as fruiting trees of economic importance. Infested plants show wilting, shoot breakage and branch dieback. *X. crassiusculus* is also included in Annex II of the Commission Implementing Regulation (EU) 2019/2072 as a non-Europeam bark beetle (EFSA 2019).

The black timber bark beetle *X. germanus* is an invasive ambrosia beetle native to East Asia, from the Kuril Islands to Vietnam, presently occurring in Europe, Russia (European and Asiatic Regions), Turkey and North America (Galko et al. 2019; Dzurenko et al. 2020; CABI 2019b; EPPO 2020). The species is polyphagous, and the wide range of its host plants includes many ornamentals, forest (broadleaf and more rarely coniferous species) and fruiting trees (Weber and McPherson 1983). Its diffusion across Europe has accelerated since 2000, probably because of climate change and the increased use of wood as packaging material (Galko et al. 2019). *X. germanus* is a xylomycetophagous species whose larvae develop freely in the maternal gallery feeding on the fungus growing on the walls; inside mycangia and maternal gallery, fungi of the genus *Ambrosiella* prevail as symbiotic organisms although many other fungi, yeasts and bacteria can be found (Mayers et al. 2015; Tuncer et al. 2018). The mechanism of host plant selection is mediated by the emission of stress-related volatiles (Ranger et al. 2010, 2015).

The economic importance of *X. germanus* is relevant in the USA, where it causes consistent damages in nurseries (Ranger et al. 2016), apple orchards (Agnello et al. 2017) and black walnut stands (Katovich, 2004); in Europe, its importance is related to outbreaks in forests (Bruge, 1995; Galko et al. 2019; Inward, 2020) and in the Mediteranean maquis (Contarini et al. 2020) but damages have been observed also in Italy on walnut (Stergulc et al. 1999) and chestnut plantations (Dutto et al. 2018). Moreover*, X. germanus* is considered a threat to the biodiversity of autoctonous Scolytinae communities (Henin and Versteirt 2004; Bouget and Noblecourt 2005), presumably because its niche overlaps with that of the indigenous species.

The three species of *Xylosandrus* may coexist in complex ecosystems with a high level of plant diversity (Contarini et al. 2020).

Taxonomic keys based on morphological characters of adults are available for *Xylosandrus* identification (Dole and Cognato 2010; Gallego et al. 2017; Garonna et al. 2012; Francardi et al. 2017), but their use may be complex as it requires a specific entomological expertise; for the larvae, identification based on morphological characters can be also difficult due to the lack of suitable keys. Therefore, the possibility of using a molecular method to univocally identify the species could be a functional tool in the operative practice, especially in areas where the insect has not been yet detected or in the niches where other *Xylosandrus* species can overlap their distribution (Francardi et al. 2017; Contarini et al. 2020). Molecular identification of *X. compactus* (Kiran et al. 2019), *X. crassiusculus* (Landi et al. 2017) and *X. germanus* (Cognato et al. 2020) has been established based on the mitochondrial cytochrome c oxidase I.

In this study, three different molecular assays aimed at identifying *X. compactus*, *X. crassiusculus*, and *X. germanus* based on qPCR with TaqMan Probe technology have been developed for a rapid and reliable identification of these invasive pests from larvae, adults, and wood chips/frass. Such tools could be very useful in phytosanitary practice where specific, and unambiguous identification methods can greatly accelerate inspection tasks conducted by Plant Protection Organizations at the national and international level, especially if they can operate with shared identification protocols.

## Materials and methods

### Biological samples

**Fig. 1:**
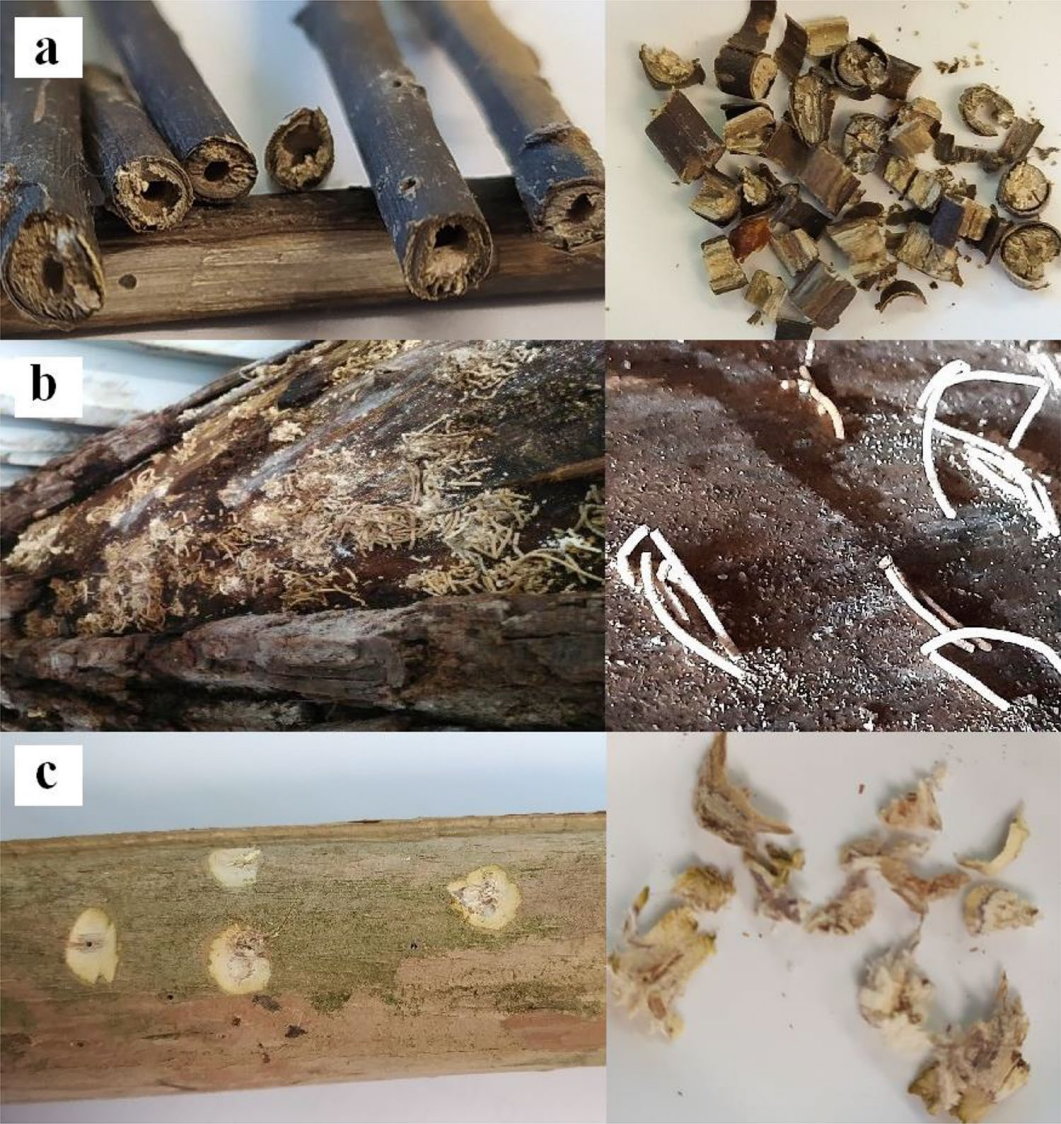
**a** *X. compactus*, **b** *X. Crassiusculus,* **c** *X. germanus*. On the left, the damage caused by the three xylophagous species on twigs or trunks; on the right, a detail of the samples used for DNA extraction

Adults and larvae of *X. compactus, X. crassiusculus* and *X. germanus* were collected during routine monitoring inspections carried out by the Phytosanitary Service in Tuscany, or by other research institutions in the respective Regions, as shown in Table 1. Larvae and adults were stored in 70% ethanol solution at room temperature, until use. The third type of samples was obtained from infested plants, in a different way for each of the three species due to the different diameters of infested twigs or trunks. For *X. compactus,* samples were collected from infested thin twigs (diameter of about 1 cm) of *Laurus nobilis* and *Rhododendron* sp., cutting small sections of wood close to the adult exit holes (Fig. 1a). For *X. crassiusculus*, samples were obtained collecting the frass from the tubes sticking out of the holes on infested logs of *Juglans nigra* (Fig. 1b) imported from USA and intercepted at the entry point of Leghorn. Adults and larvae of the wood borer inside the galleries were allowed to confirm the identification. In the case of *X. germanus*, samples were obtained in a different way, removing small wood chips (about 1 cm long) that included the last part of the insect galleries and the exit hole in an infested trunk of *Lagerstroemia indica* (Fig. 1c)*.* All these samples were stored at room temperature in the lab until use, for a period ranging from 2 to 8 months.

**Table 1:** Target and non-target samples (adults and/or larvae) and woody samples used for DNA extraction

	Internal sample number	Order	Fam/subfam	Species	Matrix/life stage
Non target samples	1	Lepidoptera	Cossidae	*Cossus cossus* Linnaeus	Frass (from *Quercus* sp)
	2			*Cossus cossus* Linnaeus	Larva
	3	Coleoptera	Cerambycidae	*Anoplophora chinensis* Förster	Frass (from *Populus* spp)
	4			*Anoplophora chinensis* Förster	Larva
	5			*Anoplophora chinensis* Förster	Larva
	6			*Anoplophora glabripennis* (Motschulsky)	Frass (from *Platanus* spp)
	7			*Anoplophora glabripennis* (Motschulsky)	Adult
	8			*Anoplophora glabripennis* (Motschulsky)	Larva
	9			*Anoplophora glabripennis* (Motschulsky)	Egg
	10			*Aromia bungii* (Faldermann)	Frass (from *Prunus* spp)
	11			*Aromia bungii* (Faldermann)	Frass (from *Prunus* spp)
	12			*Aromia bungii* (Faldermann)	Larva
	13			*Aromia moschata* (Linnaeus)	Adult
	14			*Cerambyx cerdo* Linnaeus	Adults
	15			*Cerambyx scopolii* Fuessly	Adults
	16			*Cerambyx welensii* Küster	Adult
	17			*Lepturges confluens* (Haldeman)	Larva
	18			*Monochamus galloprovincialis* Olivier	Adult
	19			*Monochamus sutor* Linnaeus	Larva
	20			*Morimus asper* (Sulzer)	Adult
	21			*Saperda carcharias* (Linnaeus)	Adult
	22			*Saperda punctata* (Linnaeus)	Adult
	23			*Saperda scalaris (*Linnaeus)	Adult
	24			*Saperda tridentata* Olivier	Larva
	25			*Saperda tridentata* Olivier	Adult
	26		Scolytinae	*Hylurgus ligniperda* (Fabricius)	Adult
	27			*Ips sexdentatus* Boern	Adult
	28			*Orthotomicus erosus* (Wollaston)	Adult
	29			*Pityophthorus juglandis* Blackman	Frass
	30			*Pityophthorus juglandis* Blackman	Adult
	31			*Pityophthorus pubescens* (Marsham)	Adult
	32			*Tomicus destruens* (Wollaston)	Adult
	33			*Xyleborinus saxesenii* Ratzeburg	Adult
	34			*Xyleborus dispar* Fabricius	Adult
	35			*Xyleborus monographus* (Fabricius)	Adult
	36		Silvanidae	*Silvanus muticus* Sharp	Larva
Target samples	37	Coleoptera	Scolytinae	*X. compactus* (Eichhoff)	Adults
	38			*X. compactus* (Eichhoff)	Larvae
	39			*X. compactus* (Eichhoff)	Sections of wood (from *Laurus nobilis*)
	40			*X. compactus* (Eichhoff)	Sections of wood (from *Laurus nobilis*)
	41			*X. crassiusculus* (Motschulsky)	Adults
	42			*X. crassiusculus* (Motschulsky)	Larvae
	43			*X. crassiusculus* (Motschulsky)	Frass (from *Juglans nigra*)
	44			*X. germanus* (Blandford)	Adults
	45			*X. germanus* (Blandford)	Larvae
	46			*X. germanus* (Blandford)	Wood chips (from *Lagerstroemia* sp)

The non-target insects (adults and/or larvae) used as comparison were part of the biomolecular collection of the Phytopathological Lab of the Phytosanitary Service of the Tuscany Region, Italy. The non-target samples used to test the specificity of the proposed method were DNAs extracted from frass of other xylophagous species. The non-target insects and frass are listed in Table 1.

### DNA extraction from target and non-target samples

Genomic DNA from all target insect and wood chips or frass samples was extracted using a modified protocol based on the CTAB one suggested by Li et al. (2008), until the addition of chloroform and the subsequent centrifugation at 11,500×*g* for 5 min. An aliquot of 600 μL of the upper phase was purified using the Maxwell^®^ RSC PureFood GMO and Authentication Kit in combination with the automated purificator MaxWell 16 (Promega, Madison, WI, USA). Details of the purification have been previously described (Rizzo et al. 2020a, b). For adult and larvae samples, single specimens were processed, for the woody samples, 500 mg of each sample were used in the extraction.

The DNA of non-target samples was extracted using the same procedure described for target samples but in different times.

The quality of the extracted DNA was assayed in qPCR after a dilution 1:20 of DNA in ddH_2_O; a dual-labeled probe targeting a highly conserved region of the 18S rDNA was used in the reaction (Ioos et al. 2009). The amplificability tests carried out in this way served as control of the extractions and allowed to verify the presence of inhibitors in relation to both the Cq detected and the slope of the relative amplification curves.

### Design of primers and probe and relative optimization

The Oligo Architect Online (Sigma-Aldrich) software was used to design the primer pairs and probes, targeting the conserved sequences of *X. compactus, X. crassiusculus* and *X. germanus* genome calculating the product size, the melting temperature and primer length. The absence of secondary structure was also considered when possible. For the development of the real-time probe method, the sequence used for each of the tested species is shown in Table 2.

**Table 2:** Primer sequences of the three *Xylosandrus* species used in this study

Organism	Description genomic region	Sequence number
*X. compactus*	BMNH 1274291 mitochondrion, partial genome	KT696209.1
*X. crassiusculus*	BMNH 1043087 mitochondrion, complete genome	KX035196.1
*X. germanus*	Mitochondrion, complete genome	KX035202.1

An *in-silico* test of the primer pairs was then performed with the BLAST^®^ (Basic Local Alignment Search Tool: http://www.ncbi.nlm.nih.gov/BLAST), software to assess the specificity of the designed primer pairs and probe. The primers/probes used in this study for real-time Probe protocol, are reported in Table 3.

**Table 3:** Primers and probe designed and used in qPCR

Species	Primer pair and probes	Lenght	Sequence	Product size (bp)
*X. compactus*	Xcomp_5117F	20	CGTGTAAGAGTTGCGTTGTC	143
	Xcomp_5229R	20	GGGTATGTTCTCCCTTGAGG	
	Xcomp_5229P	27	Cy5 - TCATTCTGAGGTGCCACTGTCATCACA- BHQ2	
*X. crassiusculus*	Xcrass_2693F	20	GCCCTTTGAATGTGGATTTG	116
	Xcrass_2808R	25	GGAGGAATAGTGTTAATTCAACATC	
	Xcrass_2721P	24	FAM - AACTCAGCTCGCCTACCATTCTCT - BHQ1	
*X. germanus*	Xgerm_3354F	18	TCCTCGTCAATTGAATGA	89
	Xgerm_3442R	19	CCACATTAGAAGGTTGAAG	
	Xgerm_3384P	22	FAM_TCGCCAGCAGAACACAGATACA_BHQ1	

**Fig. 2:**
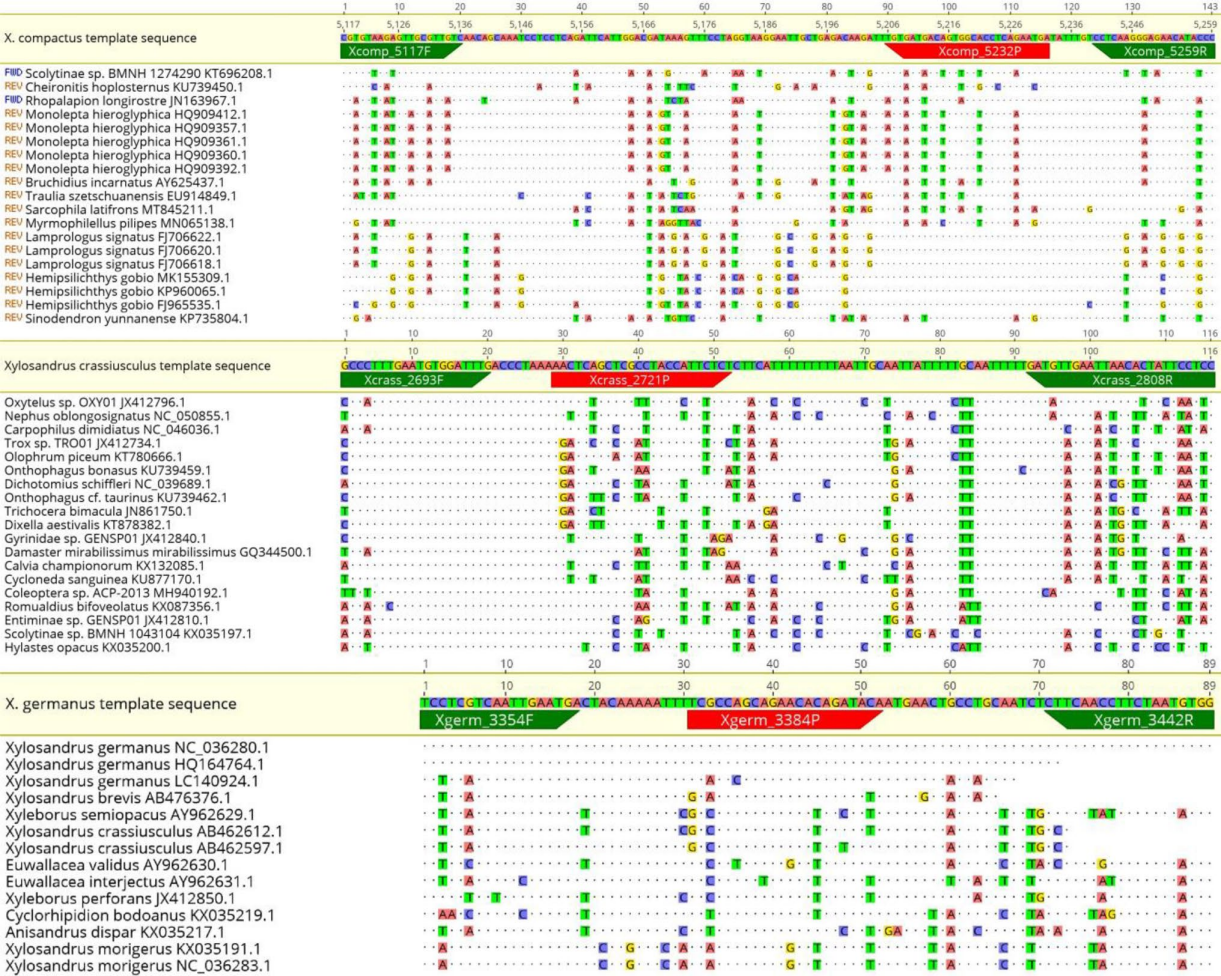
**a** *X. compactus*, **b** *X. crassiusculus*, **c** *X. germanus*. Alignment of the *in-silico* amplicons to the similar sequences of other organisms present in GenBank using the probe qPCR protocol similarity values

The in-silico specificity was further verified by searching for the most related nucleotide sequences by using the BLAST software, using as query the expected amplicons of the probe qPCR protocol. The sequences were aligned using the MAFFT program (Katoh and Standley 2013) implemented within the software Geneious^®^ 10.2.6 (Biomatters, http://www.geneious.com). The results are shown in Fig. 2a–c for the three species of *Xylosandrus* spp.

### qPCR protocols

To determine the optimal annealing temperatures, for all developed protocols, the temperature gradients from 50 to 60 °C were tested on 10 DNA samples extracted from two adults and two woody samples of *X. compactus, X. crassiusculus* and *X. germanus*. Oligos and probes were used at different concentrations: 0.2 µM, 0.3 µM and 0.4 µM each. For each target DNA sample, positive and negative amplification controls were included in each qPCR run. Samples were tested as technical duplicates and tests were repeated when unclear or contradictory results were obtained. The amplification reactions in qPCR Probe were performed with a CFX96 (Biorad, Hercules, CA) thermocycler in a final volume of 20 μL. Data obtained were analyzed with CFX Maestro 1.0 software using automatic thresholds and baselines for FAM and CY5.

In all probe assays, samples were considered positive when the correspondent qPCR curves showed a clear inflection point and an increasing kinetics, and Cq values < 35.

### Validation of the method for the qPCR Probe

Due to the possibility of using the test in routine diagnostics, performance criteria such as analytical sensitivity, analytical specificity, repeatability and reproducibility were determined. Validation was performed according to EPPO standard PM7/98 (4) 2019 for insect samples (larvae and adults) and for woody samples in each protocol assayed. In all test performed, the parameters true positives, false negatives, false positives and true negatives were considered according to EPPO standard (EPPO 2019).

The evaluation of the analytical sensitivity (limit of detection, LoD) was estimated in 1:5 serial dilutions for all qPCR protocol with TaqMan Probe for both adult and “artificial” frass samples. These last samples were obtained mixing 5 ng/µL of DNA extracted from larvae of each species to DNA from frass (on *Juglans nigra*) of a non-target species (*Pityophthorus juglandis*) diluted at 50 ng/µL. Three replicates were used using the DNA extracts diluted to 5 ng/µL. The evaluation range for all protocols studied was included between 10 ng/µL and 25.6 fg/µL. All measurements were made using the QIAxpert system (QIAGEN, Hilden, Germany).

The repeatability was tested on ten DNA samples extracted from adults and woody portions (environmental frass, sections of infested twigs and wood chips collected in the field for each of the three ambrosia beetle species as previously described). The DNA samples were diluted at a concentration of 5 ng/μl, with two independent extractions performed on each sample. The protocol of reproducibility was like the one used to test repeatability, but two different operators carried out the assays on different days.

## Results

### DNA extraction

The results of DNA extraction from the different matrices (adults, larvae and wood chips/frass) of the three species of *Xylosandrus* are shown in Table 4.

**Table 4:** Qualitative and quantitative parameters of DNA extractions from different matrices (adults, larvae and frass) for each protocol developed in this study

Parameter	Matrix	*X. compactus*	*X. crassiusculus*	*X. germanus*
DNA conc ± SD (ng/µl)	Adults	202.6 ± 16.3	168.4 ± 45.2	192.9 ± 24.6
	Larvae	321.5 ± 25.1	265.1 ± 12.5	187.3 ± 2.1
	Frass/wood chips	192.9 ± 24.6	187.3 ± 2.1	98.5 ± 12.5
(A260/280)	Adults	2.0 ± 0.1	2.0 ± 0.2	1.9 ± 0.5
	Larvae	2.0 ± 0.4	2.0 ± 0.2	2.0 ± 0.3
	Frass/wood chips	1.8 ± 0.2	1.9 ± 0.3	1.9 ± 0.2
Cq (18S rRNA)	Adults	17.9 ± 2.1	16.6 ± 1.7	16.8 ± 1.4
	Larvae	14.6 ± 0.8	14.9 ± 1.2	14.6 ± 1.4
	Frass/wood chips	21.6 ± 1.5	23.7 ± 2.4	24.9 ± 1.8

The values for the three species of *Xylosandrus* genus are reported

### Optimization of the Probe qPCR assay conditions

The optimal mix reaction for each protocol developed in this study on *Xylosandrus* species included 10 µL of 2 × QuantiNova Probe PCR Master Mix (QIAGEN, Hilden, Germany) with 0.4 µM of primers and a 0.2 µM probe concentration. The optimal annealing temperatures were equal to 55 °C, 56 °C and 58 °C for *X. compactus, X. crassiusculus,* and *X. germanus,* respectively*.* There were negligible differences in Cq values between different concentrations of primers (300 and 500 nM) or probe (150 and 250 nM).

**Fig. 3:**
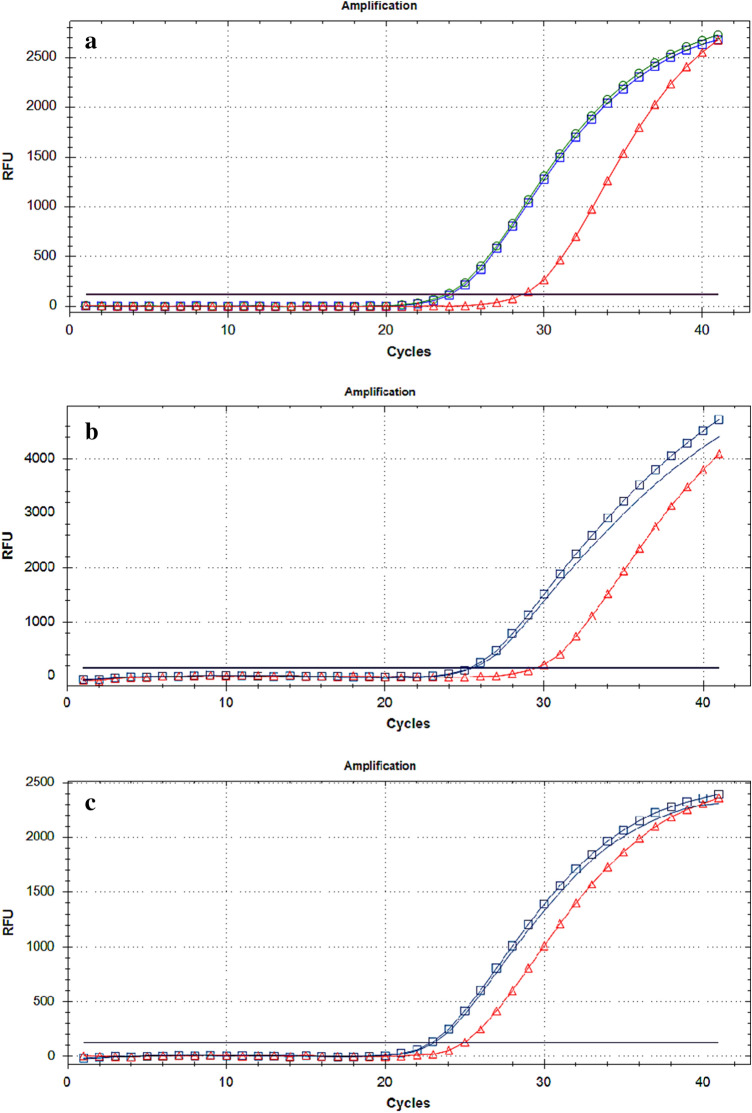
**a** *X. compactus*, **b** *X. crassiusculus*, **c** *X. germanus*. Amplification curves of qPCR Probes (squares: adults, without symbols: larvae, triangles: woody samples). DNA extracts of adults, larvae and woody samples were diluted at 10 ng/µL

The qPCR conditions consisted of an initial denaturation at 95 °C for 2 min, followed by 40 cycles of 95 °C for 10 s, and 55 °C for *X. compactus* samples, 56 °C for *X. crassiusculus* samples and 58 °C for *X. germanus* for 40 s (Fig. 3a–c).

### Validation method

The assays were inclusive for *X. compactus, X. crassiusculus* and *X. germanus,* and exclusive towards the non-target organisms tested. All target specimens were correctly identified using the specific test and no false-positive results were obtained for non-target organisms, resulting in a 100% diagnostic specificity, diagnostic sensitivity and accuracy. The test runs yielded the same qualitative results for all assayed samples and were not influenced by variation in assay conditions. The results are shown in Table 5.

**Table 5:** Analytical sensitivity (LoD) assays using 1:5 serial dilutions (from 10 ng/µL to 25.6 fg/µL) from insect adult

Samples	Dilutions 1:5	qPCR probe *X. compactus*	qPCR probe *X. crassiusculus*	qPCR probe *X. germanus*
		Cq mean ± SD	Cq mean ± SD	Cq mean ± SD
Adults	10 ng/µL	22.19 ± 0.05	22.55 ± 0.89	26.38 ± 0.28
	2.0 ng/µL	24.32 ± 0.09	24.86 ± 1.02	28.81 ± 0.07
	0.4 ng/µL	26.63 ± 0.08	28.69 ± 1.20	31.18 ± 0.03
	0.08 ng/µL	29.08 ± 0.07	30.09 ± 0.05	33.55 ± 0.14
	0.016 ng/µL	31.70 ± 0.30	32.47 ± 0.04	35.58 ± 0.27
	3.2 pg/µL	33.54 ± 0.47	34.42 ± 0.43	–
	0.64 pg/µL	–	–	–
	0.128 pg/µL	–	–	–
	25.6 fg/µL	–	–	–
Wood chips/frass	10 ng/µL	24.01 ± 0.12	22.96 ± 0.12	26.70 ± 0.14
	2.0 ng/µL	26.36 ± 0.06	25.08 ± 0.07	28.59 ± 0.06
	0.4 ng/µL	28.64 ± 0.11	27.25 ± 0.11	30.92 ± 0.10
	0.08 ng/µL	31.14 ± 0.06	29.70 ± 0.03	33.16 ± 0.03
	0.016 ng/µL	33.49 ± 0.05	32.15 ± 0.06	35.26 ± 0.48
	3.2 pg/µL	–	34.42 ± 0.23	–
	0.64 pg/µL	–	–	–
	0.128 pg/µL	–	–	–
	25.6 fg/µL	–	–	–

Cq values above 35 were considered as negative results

*Mean Cq ± SD* mean of the three threshold cycles of each dilution (Cq) ± standard deviation (SD)

Adult DNA samples of *X. compactus* and *X. crassiusculus* showed an identical LoD value of 3.2 pg/µL, while the analytical sensitivity of *X. germanus* was 0.016 ng/µL.

The woody matrix DNAs had different LoD values: in fact, the analytical sensitivity for *X. compactus* and *X. germanus* was the same (0.016 ng/µL), while for *X. crassiusculus* the LoD was 3.2 pg/µL, lower than the value observed for adult samples.

**Fig. 4:**
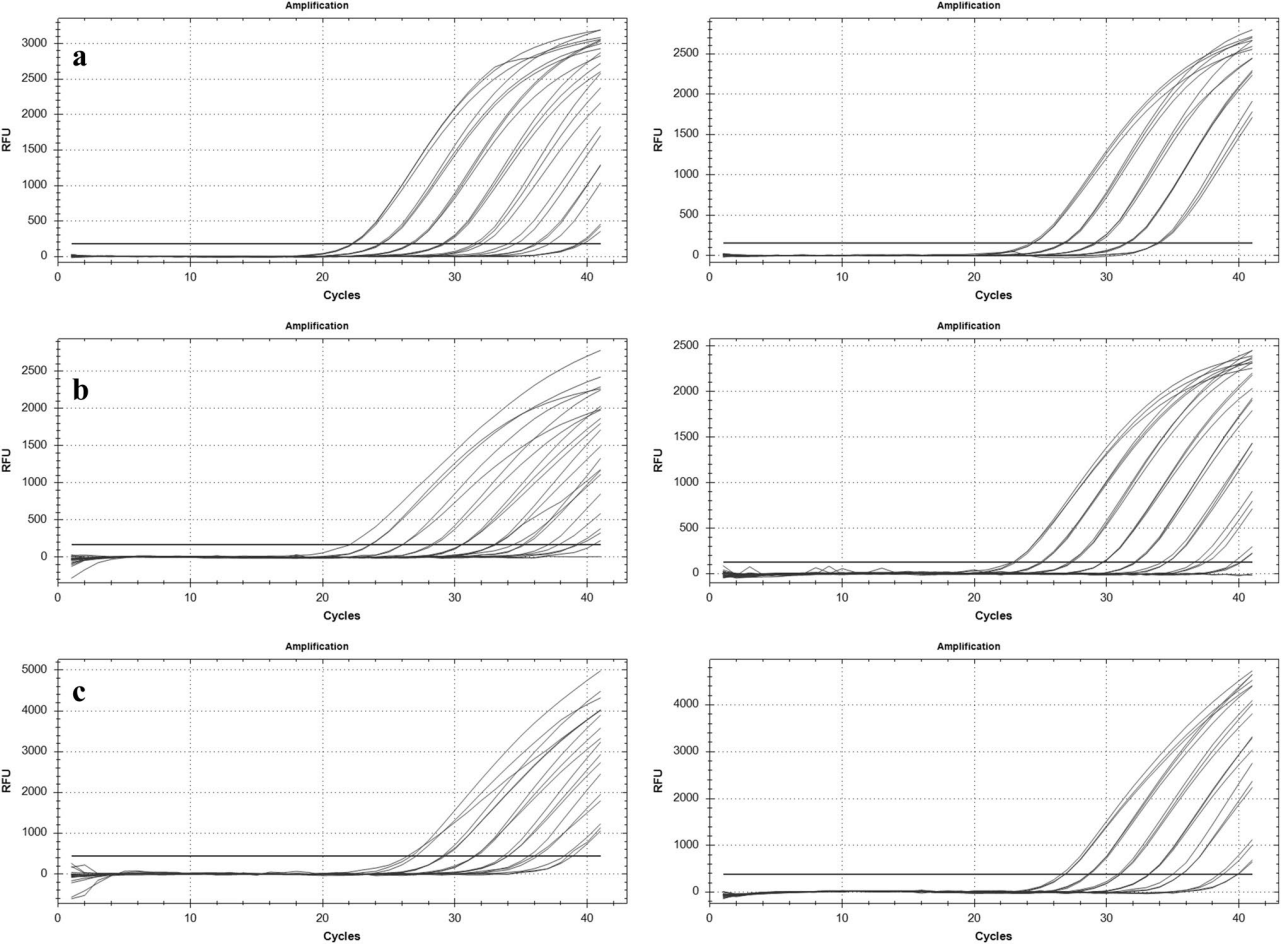
**a** *X. compactus*, **b** *X. crassiusculus,* **c** *X. germanus.* Amplification curves of qPCR Probes. Curves of amplification on the left are relative to adult samples, those on the right, to artificial frass samples

**Fig. 5:**
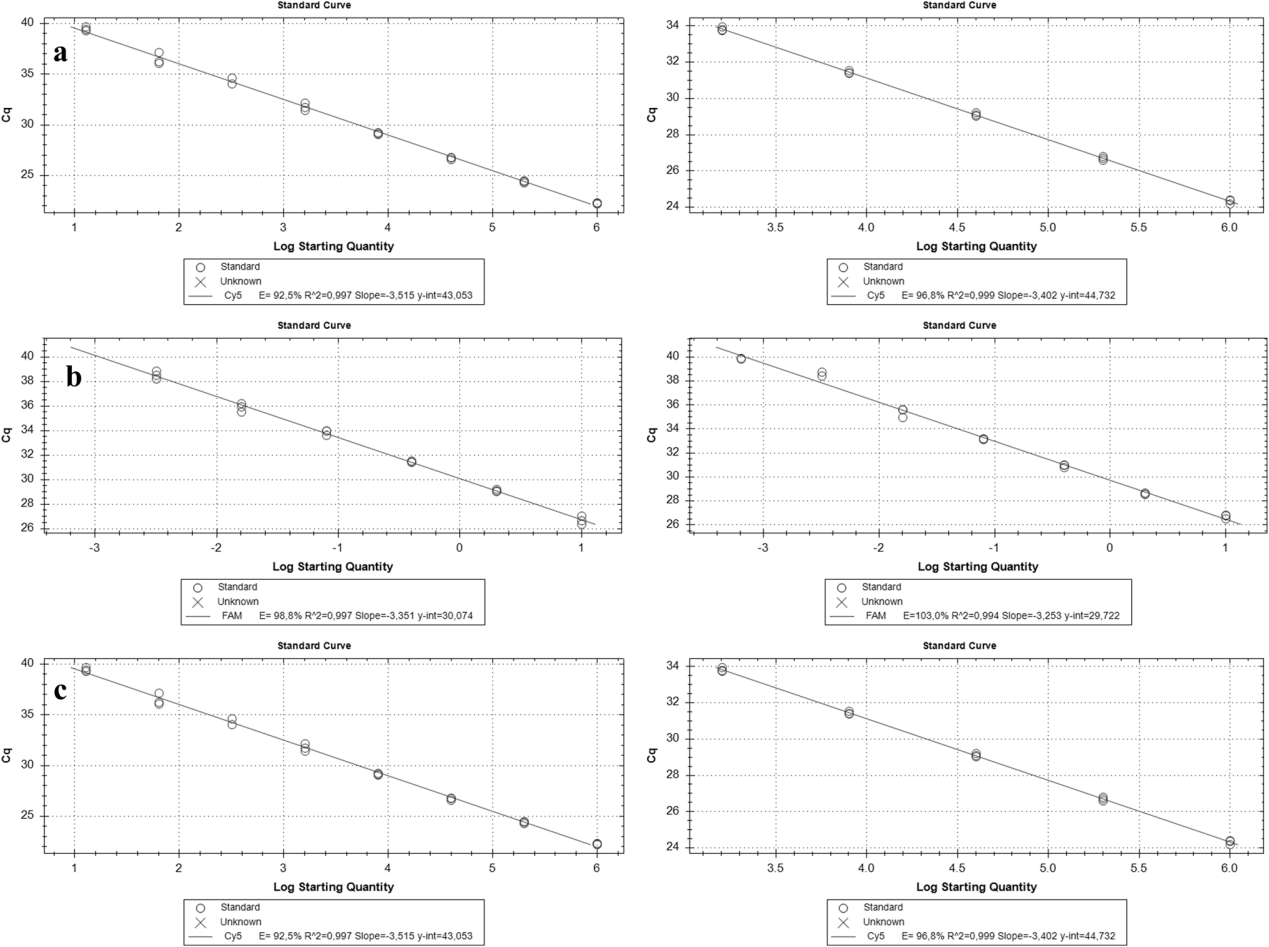
**a** *X. compactus*, **b** *X. Crassiusculus*, **c** *X. germanus*. Calibration curves of the serial diluition 1:5; adult samples (on the left) and woody samples (on the right)

The analytical sensitivity of assays for the three *Xylosandrus* species is shown in Figs. 4 and 5 where the amplification curves at the different dilutions and the respective standard curves are reported.

Repeatability and reproducibility were estimated for the three *Xylosandrus* species on adults and woody samples, which showed very low values (Teter and Steffen 2017). In fact, the repeatability values, as well as the reproducibility values measured as standard deviation (SD), varied between 0.00 and 4.11 (Table 6).

**Table 6:** Repeatability and reproducibility values (mean ± SD) on 10 replicates of DNA extract from adults and frass of the three *Xylosandrus* species; the average Cq of qPCR reaction for the three species of *Xylosandrus* genus and the correspondent standard deviations are reported

Species	Adult				Frass		
	Samples	Repeatability		Reproducibility	Repeatability		Reproducibility
		Assay 1	Assay 2		Assay 1	Assay 2	
*X. compactus*	1	20.05 ± 1.41	20.85 ± 0.18	20.89 ± 0.15	25.16 ± 4.11	28.25 ± 0.05	27.91 ± 0.23
	2	21.03 ± 0.08	20.81 ± 0.01	20.88 ± 0.13	28.01 ± 0.54	28.13 ± 0.18	28.05 ± 0.01
	3	20.96 ± 0.20	20.89 ± 0.19	20.90 ± 0.14	27.74 ± 0.87	28.06 ± 0.01	27.69 ± 0.33
	4	20.17 ± 0.77	20.54 ± 0.55	20.65 ± 0.43	27.71 ± 0.89	28.23 ± 0.34	28.04 ± 0.04
	5	20.41 ± 0.02	20.82 ± 0.13	20.82 ± 0.09	28.52 ± 0.14	27.86 ± 0.22	27.90 ± 0.09
	6	20.34 ± 1.02	21.11 ± 0.28	21.03 ± 0.24	28.61 ± 0.71	27.86 ± 0.01	27.87 ± 0.08
	7	20.83 ± 0.27	20.92 ± 0.15	20.87 ± 0.11	27.39 ± 1.05	28.18 ± 0.09	27.97 ± 0.25
	8	21.00 ± 0.10	21.06 ± 0.04	21.00 ± 0.11	28.38 ± 0.05	27.85 ± 0.33	27.76 ± 0.15
	9	21.34 ± 0.90	20.68 ± 0.06	20.78 ± 0.19	26.99 ± 1.82	28.16 ± 0.01	27.92 ± 0.23
	10	21.71 ± 1.46	20.64 ± 0.25	20.77 ± 0.29	27.73 ± 1.23	27.77 ± 0.08	28.01 ± 0.02
*X. crassiusculus*	1	22.36 ± 0.20	22.40 ± 0.08	22.31 ± 0.15	30.81 ± 0.01	30.83 ± 0.01	30.76 ± 0.11
	2	22.27 ± 0.03	22.01 ± 0.11	22.08 ± 0.15	33.2 ± 0.02	33.27 ± 0.01	32.40 ± 1.50
	3	22.11 ± 0.28	22.04 ± 0.06	22.10 ± 0.12	30.63 ± 0.00	30.63 ± 0.00	31.11 ± 0.84
	4	22.24 ± 0.17	21.85 ± 0.25	21.94 ± 0.24	30.75 ± 0.11	30.67 ± 0.00	30.66 ± 0.02
	5	22.24 ± 0.14	22.07 ± 0.13	22.03 ± 0.11	32.68 ± 0.83	32.09 ± 0.01	31.61 ± 0.82
	6	22.18 ± 0.04	22.12 ± 0.07	22.15 ± 0.07	30.27 ± 0.52	29.90 ± 0.01	31.01 ± 1.94
	7	22.93 ± 0.85	22.14 ± 0.14	22.13 ± 0.10	30.76 ± 0.12	30.84 ± 0.01	30.84 ± 0.01
	8	22.73 ± 0.86	22.25 ± 0.12	22.19 ± 0.13	33.26 ± 0.01	33.28 ± 0.01	33.28 ± 0.01
	9	22.11 ± 0.16	22.31 ± 0.05	22.25 ± 0.10	30.81 ± 0.01	30.83 ± 0.01	30.79 ± 0.07
	10	22.27 ± 0.03	22.29 ± 0.49	22.27 ± 0.35	33.26 ± 0.02	33.27 ± 0.01	32.43 ± 1.45
*X. germanus*	1	22.5 ± 0.28	22.45 ± 0.08	22.31 ± 0.15	25.63 ± 0.69	23.56 ± 0.49	24.25 ± 1.38
	2	22.5 ± 0.28	22.09 ± 0.11	22.23 ± 0.15	24.52 ± 0.96	24.53 ± 1.61	24.53 ± 2.27
	3	22.25 ± 0.04	21.99 ± 0.06	22.12 ± 0.12	25.45 ± 0.11	25.12 ± 0.08	25.23 ± 0.22
	4	21.91 ± 0.40	22.03 ± 0.25	22.12 ± 0.24	24.27 ± 0.01	24.24 ± 0.01	24.25 ± 0.02
	5	22.36 ± 0.24	22.16 ± 0.13	22.03 ± 0.11	25.81 ± 0.75	23.56 ± 0.53	24.31 ± 1.50
	6	22.34 ± 0.20	22.17 ± 0.07	22.15 ± 0.07	23.49 ± 0.58	23.56 ± 0.41	24.65 ± 1.16
	7	22.15 ± 0.05	22.24 ± 0.14	22.13 ± 0.10	29.91 ± 2.34	22.89 ± 1.65	25.23 ± 4.68
	8	23.53 ± 1.20	22.16 ± 0.12	22.19 ± 0.13	25.37 ± 0.26	24.59 ± 0.18	24.85 ± 0.52
	9	23.33 ± 1.21	22.34 ± 0.05	22.25 ± 0.10	27.64 ± 1.17	24.14 ± 0.82	25.31 ± 2.33
	10	22.00 ± 0.22	22.63 ± 0.49	22.27 ± 0.35	28.29 ± 1.39	24.13 ± 0.98	25.52 ± 2.78

## Discussion

Global trade and the use of wood as packaging material contributed to an accelerated rate of dispersal of ambrosia beetles in many parts of the world (Rassati et al. 2015; Meurisse et al. 2018). Major economic and ecological damages caused by the new invaders represent serious threats, therefore, timely species detection and identification is necessary (Blaser et al. 2018; Poland and Rassati 2019; Cognato et al. 2020).

During field monitoring and inspection activities, the availability of easy-to-handle morphological taxonomic keys could be crucial for the identification of the invasive species. In the case of the *Xylosandrus*, such taxonomic keys are available, although reserved to skilled users (Dole and Cognato 2010; Francardi et al. 2017), but their limit is the lack of morphological characters suitable to unambiguously identify the early instars of the genus, a problem widely common in xylophagous species (Pennacchio et al. 2012b; Wu et al. 2017).

In recent years, many biomolecular diagnostic tools able to discriminate the presence and to identify insect pests were developed: termites (Ide et al. 2016a), bugs and cicadids (Bouwer et al. 2014; Bracalini et al. 2015), moths (Onah et al. 2016; Kang et al. 2019), wood-boring beetles (Ide et al. 2016b; Cognato et al. 2020; Rizzo et al. 2021). The biomolecular identification methods can be efficient and allow a prompt implementation of control strategies (EFSA 2020) for the detection and eradication of quarantine pests (Augustin et al. 2012; Landi et al. 2017; Rizzo et al. 2020a).

Our study was focused on developing a sensitive and performing diagnostic molecular method able to univocally identify the three *Xylosandrus* species.

The developed qPCR Probe protocol guarantees specificity as well as robustness and can be easily performed on unidentified samples, adults or larvae, suspected to belong to the *Xylosandrus* genus (or Scolytinae subfamily). The novel approach of the current protocol lies in the possibility of using twig segments including part of the gallery (in the case of *X. compactus*) or wood chips removed around the exit hole (in the case of *X. germanus*) as a performing matrix for DNA extraction. Further investigations will be devoted to a better clarification of these opportunities, which allow the identification of a xylophagous insect in the absence of living stages and with exiguous quantities of frass. To date, we can underline the excellent yield of the DNA extraction method applied to a challenging matrix such as wood. It will be useful, in the future, to test the efficiency of this method also in different substrates such as softwoods. The proposed test is probably more expensive than the classical morphological approach, even if the use of taxonomic keys is more time consuming in many cases, as it is influenced by sample quality and available taxonomic expertise. This laboratory approach requires no more than 2 h from the beginning of DNA extraction to the evaluation of qPCR Probe results. The developed extractive protocol gave excellent results, taking about 50 min to process up to 24 single insect samples. The validation parameters provided good values in terms of specificity, sensitivity and diagnostic accuracy, confirming the more than satisfactory performance of the qPCR probe. The individual LoD (analytical sensitivity) of the method was also satisfactory, although not dependent on the starting matrix, providing evidence of its reliability (Rizzo et al. 2020a). Although the protocols suggested in this study are not economical and require good quality laboratory equipment, their optimal sensitivity even in the case of frass or wood chips samples needs to be remarked. In fact, the use of minimal quantities of these biological samples allows the identification of a species in the absence of any developmental stage (adult or larva), simplifying and speeding up the controls, and allowing the interception of a species in a higher number of cases. This tool could be of fundamental importance for phytosanitary controls at points of entry or in outbreaks of these species.

This contribution aims to be the first in a series in view of creating a molecular key for the *Xylosandrus* species present in Europe.
